# Supplementary material for: Assessing the levels of utilisation of WHO-recommended healthcare waste disposal practices at healthcare service delivery points in Uganda
Source: PLOS Glob Public Health. 2026 May 28;6(5):e0004585. doi: 10.1371/journal.pgph.0004585 (PMC13218457; doi:10.1371/journal.pgph.0004585)
Supplement: Attachment — Submitted filename: Ethical Approval_by UNCST.pdf [file pgph.0004585.s001.pdf]

Uganda National Council for Science and Technology

(Established by Act of Parliament of the Republic of Uganda)

Our Ref: HS3131ES **12 September 2023**

Christine Nalwadda
Makerere University
Kampala

Re: Research Approval: **ASSESSMENT OF SERVICE DELIVERY POINTS FOR REPRODUCTIVE HEALTH COMMODITIES IN UGANDA**

I am pleased to inform you that on **12/09/2023**, the Uganda National Council for Science and Technology (UNCST) approved the above referenced research project. The Approval of the research project is for the period of **12/09/2023** to **12/09/2024**.

Your research registration number with the UNCST is **HS3131ES**. Please, cite this number in all your future correspondences with UNCST in respect of the above research project. As the Principal Investigator of the research project, you are responsible for fulfilling the following requirements of approval:

1. Keeping all co-investigators informed of the status of the research.

2. Submitting all changes, amendments, and addenda to the research protocol or the consent form (where applicable) to the designated Research Ethics Committee (REC) or Lead Agency for re-review and approval **prior** to the activation of the changes. UNCST must be notified of the approved changes within five working days.

3. For clinical trials, all serious adverse events must be reported promptly to the designated local REC for review with copies to the National Drug Authority and a notification to the UNCST.

4. Unanticipated problems involving risks to research participants or other must be reported promptly to the UNCST. New information that becomes available which could change the risk/benefit ratio must be submitted promptly for UNCST notification after review by the REC.

5. Only approved study procedures are to be implemented. The UNCST may conduct impromptu audits of all study records.

6. An annual progress report and approval letter of continuation from the REC must be submitted electronically to UNCST. Failure to do so may result in termination of the research project.

Please note that this approval includes all study related tools submitted as part of the application as shown below:

No.	Document Title	Language	Version Number	Version Date
1	EXIT INTERVIEW TOOL	ACHOLI	TWO	28 July 2023
2	Data collection tools	Luganda	TWO (CLEAN)	27 July 2023
3	Informed Consent forms	Luganda	TWO (CLEAN)	16 August 2023
4	COVID-19 & EBOLA risk management plan for the study	ENGLISH	TWO (CLEAN)	
5	EXIT INTERVIEW TOOL	ENGLSH	TWO	28 July 2023
6	EXIT INTERVIEW TOOL	ATESO	TWO	28 July 2023
7	INFORMED CONSENT FORM	ACHOLI	TWO	28 July 2023
8	EXIT INTERVIEW TOOL	LUGBARA	TWO	28 July 2023
9	INFORMED CONSENT FORM	LUGBARA	TWO	28 July 2023
10	INFORMED CONSENT FORM	ATESO	TWO	28 July 2023
11	EXIT INTERVIEW TOOL	NGAKARAMOJONG	TWO	28 July 2023
12	EXIT INTERVIEW TOOL	RUNYAKITARA	TWO	28 July 2023
13	INFORMED CONSENT FORM	NGAKARAMOJONG	TWO	28 July 2023
14	INFORMED CONSENT FORM	RUNYAKITARA	TWO	28 July 2023
15	EXIT INTERVIEW TOOL	English	TWO	28 July 2023
16	SDP SURVEY TOOL	English	TWO	28 July 2023
17	Project Proposal	English	THREE (CLEAN)	
18	Approval Letter	English		
19	Administrative Clearance	English		

Yours sincerely,

Hellen Opolot
For: Executive Secretary
UGANDA NATIONAL COUNCIL FOR SCIENCE AND TECHNOLOGY

LOCATION/CORRESPONDENCE

Plot 6 Kimera Road, Ntinda
P.O. Box 6884
KAMPALA, UGANDA

COMMUNICATION

TEL: (256) 414 705500
FAX: (256) 414-234579
EMAIL: info@uncst.go.ug
WEBSITE: http://www.uncst.go.ug
