# Supplementary material for: Assessing the levels of utilisation of WHO-recommended healthcare waste disposal practices at healthcare service delivery points in Uganda
Source: PLOS Glob Public Health. 2026 May 28;6(5):e0004585. doi: 10.1371/journal.pgph.0004585 (PMC13218457; doi:10.1371/journal.pgph.0004585)
Supplement: Attachment — Submitted filename: Response to reviewers.docx [file pgph.0004585.s004.docx]

**PGPH-D-25-00169**

PLOS Global Public Health

**Assessing the levels of utilisation of WHO-recommended healthcare waste disposal practices at healthcare service delivery points in Uganda**

**Dear Sir/Madam,**

We greatly appreciate the thorough review of our manuscript and the invaluable feedback. Below, we outline the concerns raised and how we have addressed them.

**Response to the reviewer’s comments**

| No. | **Section of the manuscript** | **Comments/ concerns** | **Action taken** |
| --- | --- | --- | --- |
| 1. | Manuscript title | A change of the manuscript title from “Magnitude of utilisation of recommended healthcare waste disposal practices at healthcare service delivery points in Uganda” to “**Assessing the levels of utilisation of WHO-recommended healthcare waste disposal practices at healthcare service delivery points in Uganda**“ | Thank you very much for these good suggestions on the revision of our manuscript title. It has been revised to “**Assessing the levels of utilisation of WHO-recommended healthcare waste disposal practices at healthcare service delivery points in Uganda** “ |
| 2. | Author affiliations | Write this in the same pattern as the correspondent author’s information, with the P. O. Box address, Names of Department, School or Faculty or College, University, Town, and City. | Thank you very much for this comment. This has been adjusted as per your guidance. |
| 3. | **Abstract**  Lines 39-40: “Inadequate healthcare waste management (HCWM) poses significant challenges to public health, environmental sustainability and community wellbeing”. | Considers replacing the word ‘significant’ with ‘substantial’ since the former is heavily linked with p-values less than 0.05 and is more statistical than practical. Consider this suggestion throughout the manuscript. | Thank you very much for this comment. This was an error; we have adjusted this throughout the manuscript |
| 4. | **Lines 43-44:** “We assessed the level of use of recommended healthcare waste disposal practices at healthcare service delivery points (HSDPs) in Uganda”. | Anytime you use ‘recommended healthcare waste disposal practices’, readers would like to know which specific recommendation you are referring to. It is important to state this upfront and throughout the manuscript. The use of HSDPs is not a standard abbreviation; writers may rather consider using ‘healthcare waste disposal practices’ (HWDP) at ‘healthcare service delivery points’ (HSDP) in Uganda. | Thank you very much for your guidance. This has been revised, and it is now stated as the WHO-recommended HWDP.  The abbreviation for ‘healthcare service delivery points’ has also been revised to **HSDP** |
| 5. | Lines 46-47: “We conducted a cross-sectional survey among 681 HSDPs randomly selected from the 15 sub-regions of Uganda between September and December 2023”. | Remove the article ‘the’ since readers may not already be aware of those ‘15 sub-regions…’ | This was an oversight article ‘the’ has been removed. |
| 6. | Lines 49-50: “The sample size of the HSDPs was determined using the standard sampling methodology provided by the United Nations Population Fund (UNFPA)”. | Readers would like to know the actual sample size calculated, and this is stated within the abstract. | The sample size of the 681 HSDP was determined using the standard sampling methodology |
| 7. | Lines 55-57: “We found notable variations in the use of recommended HCW disposal practices, for instance, the use of incineration and centralised collection by a special agency across different facility levels and regions | What does ‘notable’ mean? Is there a value from the findings depicting ‘notable’? This must be stated. | This was an oversight. We have improved wording to: |
| 8. | Lines 65-66: “Additionally, urban facilities were more likely to use regular garbage disposal (59.4%, 149/251) compared to rural facilities (19.5%, 83/425)”. | It will be critical to state the magnitude of likelihood of regular garbage disposal practices in urban facilities in comparison with rural counterparts, along with the corresponding p-values. This will mean conducting further inferential analysis, like regression, to determine odds ratios, etc. | Our research did not set out to study factors associated with the outcome. Therefore, we did not run any regression models for inferential statistics and present only the magnitudes (proportions) descriptively.  We acknowledge the limitations of descriptive research, but this information could serve as a basis for further research. |
| 9. | Lines 68-72: “The findings from this nation-wide assessment of healthcare disposal practices highlight critical gaps in the use of recommended healthcare waste disposal practices at HSDPs in Uganda, with large variations by facility level, location, and region. These gaps require targeted interventions, including the review and enforcement of existing guidelines and regulations on healthcare waste management”. | It will be important for the study conclusions to align with the stated objectives and key findings, as this is currently not the case. | Thank you for your review. This was an oversight, and it has been revised |
| 10. | Methods | Information on study participants and their characteristics needs to be provided. Who were the ‘HSDP’ managers?. An additional strength of this study will be to identify covariates and outcome variables and inferentially measure associations between key variables, thereby broadening its appeal and strengthening its rigour. This is because using only descriptive analysis narrows the scope of this journal. Providing the study site and locations in a global perspective by writing the GPS coordinates and including maps that link the selected study settings will be more appropriate. | Health Service Delivery Point (HSDP) managers included (HSDP incharges, Clinicians and IPC focal point persons).  Major outcomes: levels of utilisation of the WHO-recommended HWDP at the HSDP as incorporated in line 180 to 183  Our research did not set out to study factors associated with the outcome. Therefore, we did not run any regression models for inferential statistics, and we only present the magnitude (proportions) descriptively.  We did not have a research objective that needed us to use GPS coordinates data, so we will avoid the objective-methods- and results discordance. Also, we lack **explicit Consent**: we can not publish GPS data if we did not obtain separate, explicit consent from participants for the collection and potential sharing of these specific points. |
| 11. | Results | The background and demographic features of the 681 of 762 ‘HSDPs’ surveyed need to be presented as Table 1, segregated by **sex, age, location**, etc., and the details used to ascertain possible effects on waste disposal practices, etc. | Thank you so much for this comment.  We collected facility-level data, which included the facility characteristics. Although the interviews at the facilities were conducted with health care professionals, we did not take their individual characteristics like age, sex, location, etc. So, we cannot disaggregate by these characteristics. |
| 12. | Discussion and Conclusions | Will then be revised to reflect the current findings. | This has been revised to align with the proposed adjustments. |

We look forward to having this manuscript published in the PLOS Global Public Health journal.

Yours’ sincerely

**Dr. Christine Nalwadda**
